# Risk and prognosis of secondary bladder cancer after radiation therapy for pelvic cancer

**DOI:** 10.3389/fonc.2022.982792

**Published:** 2022-08-24

**Authors:** Shuofeng Li, Ran Wei, Guanhua Yu, Hengchang Liu, Tianli Chen, Xu Guan, Xishan Wang, Zheng Jiang

**Affiliations:** Department of Colorectal Surgery, National Cancer Center/National Clinical Research Center for Cancer/Cancer Hospital, Chinese Academy of Medical Sciences and Peking Union Medical College, Beijing, China

**Keywords:** pelvic cancer, secondary bladder cancer, radiotherapy, prognostic factor, overall survival (OS)

## Abstract

**Background:**

Radiation therapy (RT) is a crucial modality for the local control of pelvic cancer (PC), but the effect of pelvic RT on the development of secondary malignancy is still unclear. This study aimed to identify the relationship between radiation therapy received for the treatment of primary PC and subsequent secondary bladder cancer (SBC).

**Methods:**

The Surveillance, Epidemiology, and End Results (SEER) database (from 1975 to 2015) was queried for PC. Fine-gray competing risk regression and Cox regression analyses were employed to assess the cumulative incidence of SBC. Poisson regression and multiple primary standardized incidence ratios (SIR) were used to evaluate the radiotherapy-associated risk for patients receiving RT. Subgroup analyses of patients stratified by latency time since PC diagnosis, calendar year of PC diagnosis stage, and age at PC diagnosis were also performed. Overall survival (OS) was compared among different treatment groups with SBC by Kaplan–Meier analysis.

**Results:**

A total of 318,165 observations showed that the primary cancers were located in pelvic cavity, 256,313 patients did not receive radiation therapy (NRT), 51,347 patients who underwent external beam radiation therapy (EBRT), and 10,505 patients receiving a combination of EBRT and brachytherapy (EBRT–BRT) who developed SBC. Receiving two types of radiotherapy was strongly consistent with a higher risk of developing SBC for PC patients in Fine-Gray competing risk regression (NRT vs. EBRT, adjusted HR= 1.71, 95% CI: 1.54-1.90, P<0.001; NRT vs. EBRT–BRT, adjusted HR= 2.16, 95% CI: 1.78-2.63, P<0.001). The results of the dynamic SIR and Poisson regression analysis for SBC revealed that a slightly increased risk of SBC was observed after RT in the early latency and was significantly related to the variations of age at PC diagnosis and decreased with time progress. For OS, the SBC after NRT, SBC after EBRT, and SBC after EBRT-BRT of 10-year survival rates were 37.9%, 29.2%, and 22.2%, respectively.

**Conclusion:**

Radiotherapy for primary PC was associated with higher risks of developing SBC than patients unexposed to radiotherapy. Different pelvic RT treatment modalities had different effects on the risk of SBC.

## Introduction

Radiation therapy (RT) is one of the basic modalities in the treatment of pelvic tumors, including malignancies in the rectum, cervix, ovary, and so on. RT may help decrease the risk of tumor recurrence and significantly improve the prognosis (1). Patients with improved survival suffer from long-term risks after receiving RT, such as the development of second primary malignancies (SPM) (2). The studies of the Surveillance, Epidemiology, and End Results (SEER) database noted that the incidence rate of subsequent primary malignancies in cancer survivors was approximately 14% higher than that in the general population, which was related to the first cancer treatment and genetic factors (3, 4). Therefore, the long-term adverse events of radiotherapy should be carefully considered (5–7).

During RT, high doses of ionizing radiation are delivered, which is a form of high-energy electromagnetic radiation reaching deeper internal body structures and eventually causing apoptosis (8, 9). However, RT increases the risk of developing radiation-induced complications in normal tissues and may promote progressive changes in the extracellular matrix and the development of a vascular reaction, which could increase the risk of secondary tumors in the irradiated field (4, 10, 11). The bladder is usually within the field of irradiation in the pelvis and is exposed to more radiation than organs in the non-pelvic area (12). Bladder cancer as a SPM is increasingly common, but its risks are poorly understood (11, 13, 14). Secondary cancer development is a multifactorial process, and the relationship between pelvic radiation therapy and subsequent secondary bladder cancer (SBC) remains unclear. In addition, it is not known what kind of radiotherapy regimens might be related to SBC development.

In this study, the data obtained from the SEER registries with more than three decades of follow-up were used. We comprehensively analyze the incidence and latency period of subsequent bladder cancer following primary pelvic cancer (PC) irradiation.

## Methods

### Database source

The SEER database is currently the largest publicly available cancer database, covering approximately 34.6% of the U.S. cancer population. In this study, we used SEER*stat software, version 8.3.9 (http://seer.cancer.gov/seerstat/), to download patient data of diagnosed PC from 9 registries in the SEER database between January 1, 1975, and December 31, 2015. Access to and use of the data in the SEER database does not require informed consent from patients because the data and information were anonymized and deidentified before release. The study was approved by the Ethics Committee of the National Cancer Center/National Clinical Research Center for Cancer/Cancer Hospital, the Chinese Academy of Medical Sciences and Peking Union Medical College (Beijing, China).

### Study population

In this study, according to SEER’s International Classification of Disease (ICD-O-3), PCs were chosen at six sites that are routinely treated with radiotherapy (rectum and rectosigmoid cancer, cervix uteri cancer, corpus uteri cancer, ovary cancer, prostate cancer, and anus, anal canal and anorectum cancer). This study collected information on cancer patient demographic profiles and cancer incidence, including age, sex, race, primary tumor site, second primary cancers, stage, grade, limited data on clinicopathological, treatment profiles, and survival data.

### Treatment interventions

The SEER program collected information on the first course of treatment. According to the initial treatment modality of PC, patients could be classified into two groups. The RT group was composed of patients with PC who were receiving two types of radiotherapy, including external beam radiation therapy (EBRT) and a combination of external beam radiation therapy with brachytherapy involving implants or isotopes (EBRT–BRT). The no radiotherapy (NRT) group was composed of patients without two types of radiotherapy. Patients receiving brachytherapy, radioisotopes, or combination RT were censored to decrease the bias caused by different types of RT.

### Survival outcomes

The primary outcome of this research was to investigate the risk of developing SBC more than one year after treatment for PC. The SEER program has eliminated the involvement of recurrent PC disease according to the ICD-O-3 guidelines. The secondary outcome was to estimate the 10-year overall survival (OS), which was defined as the time from the start of randomization until death due to any cause.

### Statistical analysis

Fisher’s exact test and χ^2^ tests were employed to compare categorical data. Fine-Gray competing risk regression analysis was used to evaluate the cumulative incidence of SBC development. SBC was considered the event, and non-SBC or all-cause death was defended as competing events. Cox proportional hazard regression analysis was performed with SBC to evaluate the hazard ratio (HR) and 95% confidence interval (95% CI) of developing SBC after PC. The multivariable Cox analysis was established by employing a backward selection procedure with variables with 2-sided P<0.05 in univariable studies, which were considered statistically significant and included in multivariable analyses. The radiotherapy-associated risk (RR) was calculated by Poisson regression analysis with the relative risk and 95% CI of SBC development for PC patients receiving radiotherapy compared with those not receiving radiotherapy. These analyses were performed with R software, version 3.5.3. In addition, the standardized incidence ratio (SIR) and 95% CI were also estimated by Poisson regression analysis. The definition of SIR was the ratio of observed developing SBC among PC survivors in the U.S. general population. The SIR were estimated with SEER*Stat 8.3.9. Both SIR and RR were adjusted for age at PC diagnosis, race, sex, and the calendar year of PC diagnosis. They were stratified by latency time since PC diagnosis, age at PC diagnosis, and calendar year of PC diagnosis. The Kaplan–Meier method was used to determine 10-year OS for SBC, and survival differences were calculated by the log-rank test.

## Results

### Patient characteristics

**Figure 1:**
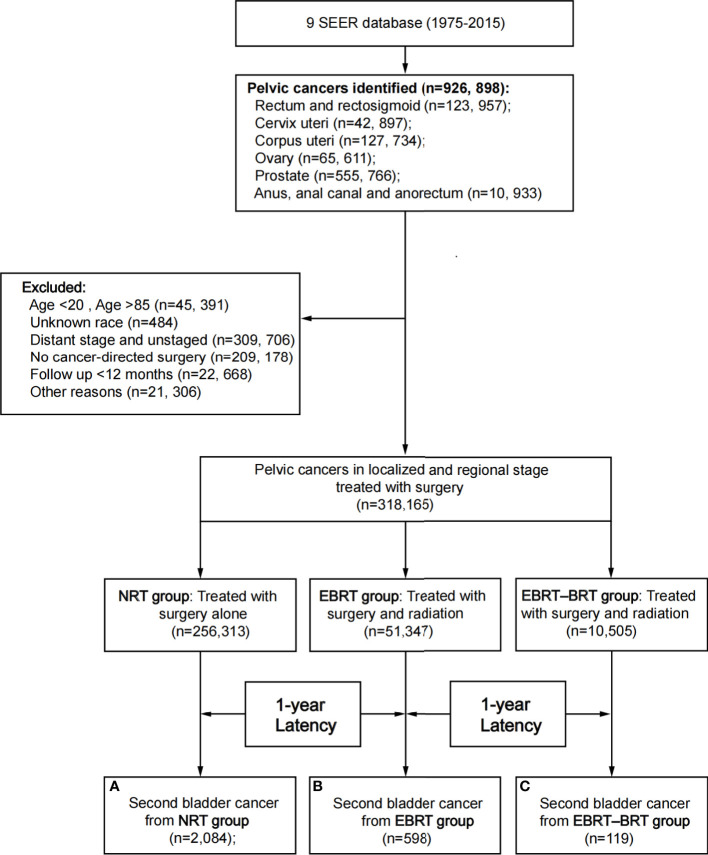
Flow diagram. SEER, Surveillance, Epidemiology and End Results; RT, radiation therapy; NRT, no radiation therapy; EBRT, external beam radiation therapy; EBRT–BRT, external beam radiation therapy with brachytherapy involving implants or isotopes.

A total of 318,165 patients with PC were identified in the study; 256,313 patients did not receive RT, 61,852 patients received RT, 51,347 of whom were treated with EBRT only, and 10,505 with EBRT–BRT (**Table 1**, **Figure 1**). PC was located in the rectum and rectosigmoid (22.54%), cervix uteri (6.35%), corpus uteri (25.53%), ovary (4.48%), prostate (39.86%), anus, anal canal, and anorectum (1.23%).

**Table 1:** Comparisons of Baseline Characteristics of Patients with PC by Treatment Modality.

	Total (N = 318165)	NRT (N = 256313)	RT (N = 61852)	RT Type	
				EBRT (n = 51347)	EBRT–BRT (n = 10505)
**Age at PC diagnosis, No. (%)**					
20-49	48141 (15.13)	37610 (14.67)	10531 (17.03)	8105 (15.78)	2426 (23.09)
50-69	199045 (62.56)	163265 (63.70)	35780 (57.85)	30080 (58.58)	5700 (54.26)
70-84	70979 (22.31)	55438 (21.63)	15541 (25.13)	13162 (25.63)	2379 (22.65)
**Year of PC diagnosis, No. (%)**					
1975-1984	41287 (12.98)	31183 (12.17)	10104 (16.34)	7541 (14.69)	2563 (24.40)
1985-1994	44742 (14.06)	32076 (12.51)	12666 (20.48)	10020 (19.51)	2646 (25.19)
1995-2004	108658 (34.15)	88801 (34.65)	19857 (32.10)	16668 (32.46)	3189 (30.36)
2005-2015	123478 (38.81)	104253 (40.67)	19225 (31.08)	17118 (33.34)	2107 (20.06)
**Sex, No. (%)**					
Female	148559 (46.69)	112291 (43.81)	36268 (58.64)	26450 (51.51)	9818 (93.46)
Male	169606 (53.31)	144022 (56.19)	25584 (41.36)	24897 (48.49)	687 (6.54)
**Race, No. (%)**					
White	265961 (83.59)	214760 (83.79)	51201 (82.78)	42588 (82.94)	8613 (81.99)
Black	28600 (8.99)	23231 (9.06)	5369 (8.68)	4344 (8.46)	1025 (9.76)
Other	23604 (7.42)	18322 (7.15)	5282 (8.54)	4415 (8.60)	867 (8.25)
**Tumor grade, No. (%)**					
Grade I/II	192565 (60.52)	156877 (61.21)	35688 (57.70)	30417 (59.24)	5271 (50.18)
Grade III/IV	83387 (26.21)	64853 (25.30)	18534 (29.97)	15129 (29.46)	3405 (32.41)
Unknow	42213 (13.27)	34583 (13.49)	7630 (12.34)	5801 (11.30)	1829 (17.41)
**Tumor stage, No. (%)**					
Localized	139504 (43.85)	115759 (45.16)	23745 (38.39)	18612 (36.25)	5133 (48.86)
Regional	51838 (16.29)	23444 (9.15)	28394 (45.91)	23647 (46.05)	4747 (45.19)
Localized/regional (Prostate cases)	126823 (39.86)	117110 (45.69)	9713 (15.70)	9088 (17.70)	625 (5.95)
**Tumor size, No. (%)**					
<5 cm	25631 (8.06)	23760 (9.27)	1871 (3.02)	1685 (3.28)	186 (1.77)
≥5 cm	44063 (13.85)	31140 (12.15)	12923 (20.89)	11365 (22.13)	1558 (14.83)
Unknown	248471 (78.10)	201413 (78.58)	47058 (76.08)	38297 (74.58)	8761 (83.40)
**Tumor site, No. (%)**					
Rectum and Rectosigmoid	71717 (22.54)	47500 (18.53)	24217 (39.15)	24120 (46.97)	97 (0.92)
Cervix Uteri	20215 (6.35)	14645 (5.71)	5570 (9.01)	2586 (5.04)	2984 (28.41)
Corpus Uteri	81229 (25.53)	61852 (24.13)	19377 (31.33)	12625 (24.59)	6752 (64.27)
Ovary	14265 (4.48)	13617 (5.31)	648 (1.05)	634 (1.23)	14 (0.13)
Prostate	126823 (39.86)	117110 (45.69)	9713 (15.70)	9088 (17.70)	625 (5.95)
Anus, Anal Canal and Anorectum	3916 (1.23)	1589 (0.62)	2327 (3.76)	2294 (4.47)	33 (0.31)
**Chemotherapy, No. (%)**					
No	280462 (88.15)	243506 (95.00)	36956 (59.75)	28322 (55.16)	8634 (82.19)
Yes	37703 (11.85)	12807 (5.00)	24896 (40.25)	23025 (44.84)	1871 (17.81)

PC, pelvic cancers; NRT, no radiation therapy; RT, radiation therapy; EBRT, external beam radiation therapy; EBRT–BRT, external beam radiation therapy with brachytherapy involving implants or isotopes.

After a minimum latency of 1 year from primary PC diagnosis, a total of 2,801 patients developed SBC. Among patients who underwent RT, 717 (1.16%) went on to develop SBC, including 598 (1.16%) patients in the EBRT group and 119 (1.13%) patients in the EBRT–BRT group who developed SPC. In the NRT group, 2084 (0.81%) patients developed SBC. The above data show that, compared with the NRT group, a greater proportion of patients who received EBRT and EBRT–BRT for their primary PC developed an SBC, and no difference was found between the two types of radiotherapy groups (**Supplementary Table 1**).

### Cumulative incidences of SBC

All variables identified in **Table 1** were selected for univariable Fine-Gray competing risk regression and Cox regression analysis to estimate the risk of developing SBC (**Table 2**, **Supplementary Table S2**). In the univariable analysis, factors including age at PC diagnosis, year at PC diagnosis, sex, race, tumor grade, tumor stage, tumor size, tumor site, chemotherapy, and radiation were associated with a higher risk of developing SBC in Fine-Gray competing risk regression analysis. Factors including age at PC diagnosis, year at PC diagnosis, sex, race, tumor grade, tumor stage, tumor site and radiation were associated with a higher risk of developing SBC in Cox regression analysis. These statistically significant parameters in the univariate analysis were included in the multivariate model.

**Table 2:** Univariable and Multivariable Competing Risk Regression Analysis of Risk of Developing SBC in PC Patients.

Characteristic	Univariable analysis		Multivariable analysis	
	HR (95% Cl)	P-value	HR (95% Cl)	P-value
**Age at PC diagnosis**				
20-49	Ref		Ref	
50-69	3.48 (2.94-4.12)	<0.001	2.36 (1.96-2.85)	<0.001
70-84	4.25 (3.56-5.07)	<0.001	3.03 (2.50-3.67)	<0.001
**Year of PC diagnosis**				
1975-1984	Ref		Ref	
1985-1994	0.96 (0.84-1.10)	0.550	0.93 (0.81-1.06)	0.270
1995-2004	1.55 (1.40-1.72)	<0.001	0.82 (0.71-0.95)	<0.001
2005-2015	1.03 (0.91-1.17)	0.660	0.58 (0.49-0.69)	<0.001
**Sex**				
Female	Ref		Ref	
Male	2.97 (2.73-3.22)	<0.001	2.93 (2.45-3.51)	<0.001
**Race**				
White	Ref		Ref	
Black	0.60 (0.51-0.70)	<0.001	0.56 (0.48-0.66)	<0.001
Other	0.51 (0.42-0.62)	<0.001	0.58 (0.48-0.71)	<0.001
**Tumor grade**				
Grade I/II	Ref		Ref	
Grade III/IV	1.15 (1.05-1.25)	<0.001	1.05 (0.96-1.15)	0.340
Unknow	0.64 (0.56-0.72)	<0.001	1.00 (0.88-1.15)	0.950
**Tumor stage**				
Localized	Ref		Ref	
Regional	1.24 (1.11-1.40)	<0.001	0.85 (0.74-0.97)	0.015
Localized/regional (Prostate cases)	2.37 (2.18-2.57)	<0.001	NA	NA
**Tumor size**				
<5 cm	Ref		Ref	
≥5 cm	0.68 (0.53-0.87)	0.002	0.94 (0.73-1.22)	0.630
Unknown	1.18 (0.97-1.42)	0.096	1.02 (0.84-1.26)	0.830
**Tumor site**				
Rectum and Rectosigmoid	Ref		Ref	
Cervix uteri	0.40 (0.32-0.50)	<0.001	1.30 (0.98-1.74)	0.068
Corpus uteri	0.58 (0.52-0.66)	<0.001	1.12 (0.93-1.36)	0.240
Ovary	0.32 (0.24-0.43)	<0.001	0.93 (0.67-1.31)	0.690
Prostate	1.56 (1.43-1.71)	<0.001	1.31 (1.12-1.53)	<0.001
Anus, anal canal and anorectum	0.80 (0.56-1.16)	0.250	0.96 (0.66-1.40)	0.830
**Chemotherapy**				
No	Ref		Ref	
Yes	0.77 (0.67-0.87)	<0.001	0.89 (0.75-1.05)	0.170
**Radiation**				
No	Ref		Ref	
EBRT	1.38 (1.26-1.51)	<0.001	1.71 (1.54-1.90)	<0.001
EBRT–BRT	1.19 (1.00-1.42)	0.057	2.16 (1.78-2.63)	<0.001

Fine-Gray competing risk regression analyses were used to calculate the hazard ratio (HR) and 95% confidence interval (CI) for SBC in pelvic cancers patients treated with RT versus patients not treated with RT.

PC, pelvic cancers; HR, hazard ratio; CI, confidence interval; EBRT, external beam radiation therapy; EBRT–BRT, external beam radiation therapy with brachytherapy involving implants or isotopes.

**Figure 2:**
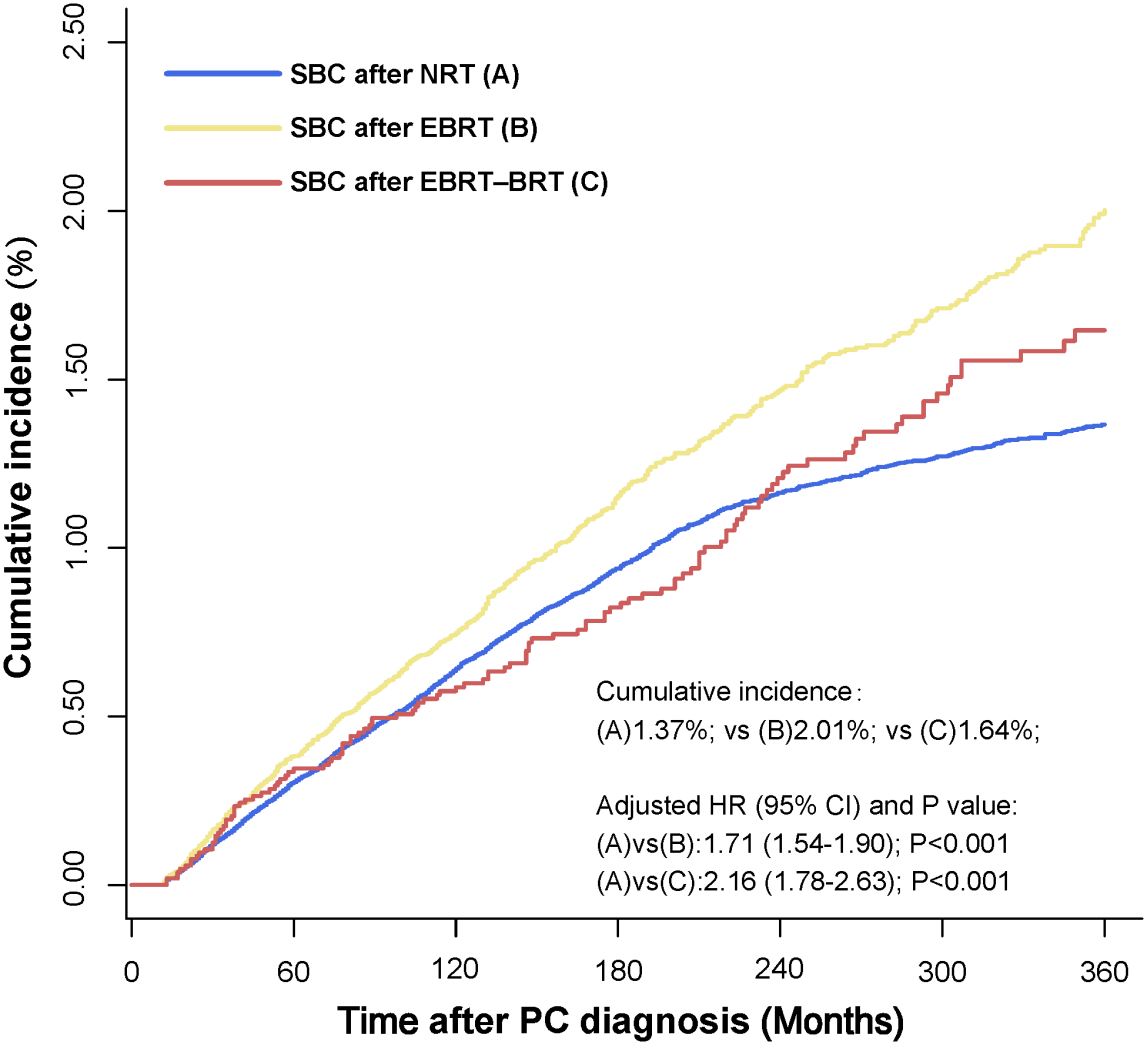
Comparisons of cumulative incidence of secondary bladder cancer (SBC) between patients who received radiation therapy (RT) and patients who did not receive RT. P values were calculated with the Fine-Gray test. PC, pelvic cancers; SBC, secondary bladder cancer; HR, hazard ratio; CI, confidence interval; NRT, no radiation therapy; EBRT, external beam radiation therapy; EBRT–BRT, external beam radiation therapy with brachytherapy involving implants or isotopes.

In the multivariate analysis, factors including age at PC diagnosis, year at PC diagnosis, sex, race, tumor stage, tumor site, and radiation had a higher risk of developing SBC in the Fine-Gray competing risk regression analysis. The factors included age at PC diagnosis, year at PC diagnosis, sex, race, tumor grade, and radiation with a higher risk of developing SBC in Cox regression analysis. Receiving two types of radiotherapy was strongly consistent with a higher risk of developing SBC for PC patients in Fine-Gray competing risk regression (NRT vs. EBRT, adjusted HR= 1.71, 95% CI: 1.54-1.90, P<0.001; NRT vs. EBRT–BRT, adjusted HR= 2.16, 95% CI: 1.78-2.63, P<0.001) and Cox regression analysis (NRT vs. EBRT, adjusted HR= 1.75, 95% CI: 1.59-1.94, P<0.001; NRT vs. EBRT–BRT, adjusted HR= 2.33, 95% CI: 1.91-2.84, P<0.001). The cumulative incidence of SBC after NRT, SBC after EBRT and SBC after EBRT-BRT were 1.37%, 2.01%, and 1.64%, respectively (**Figure 2**).

**Figure 3:**
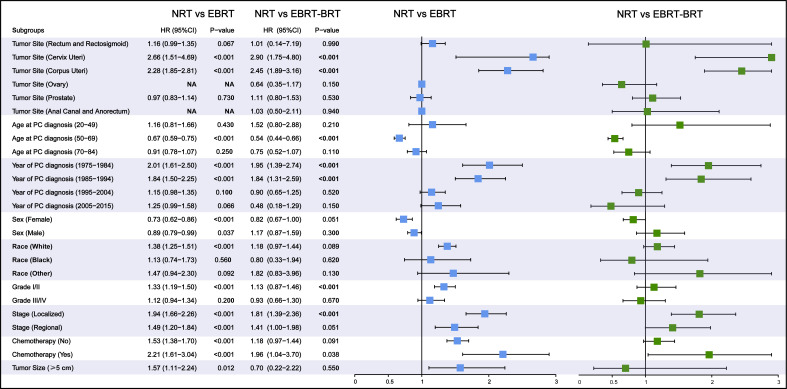
Subgroup analyses of competing risk regression for the risk of developing secondary bladder cancer (SBC). PC, pelvic cancers; SBC, secondary bladder cancer; HR, hazard ratio; CI, confidence interval; NRT, no radiation therapy; RT, radiation therapy; EBRT, external beam radiation therapy; EBRT–BRT, external beam radiation therapy with brachytherapy involving implants or isotopes.

Subgroup analyses were performed to further evaluate the risk of developing SBC by competing risk regression. We found that the increased risk associated with RT was noted in most subgroups. In analyses of each type of PC, receiving two types of radiotherapy could significantly increase risks in cervix uteri cancer (NRT vs. EBRT, adjusted HR= 2.66, 95% CI: 1.51-4.69, P<0.001; NRT vs. EBRT–BRT, adjusted HR= 2.90, 95% CI: 1.75-4.80, P<0.001) and corpus uteri cancer (NRT vs. EBRT, adjusted HR= 2.28, 95% CI: 1.85-2.81, P<0.001; NRT vs. EBRT–BRT, adjusted HR= 2.45, 95% CI: 1.89-3.16, P<0.001) (**Figure 3**).

### Dynamic risk and incidence evaluation for SBC

**Figure 4:**
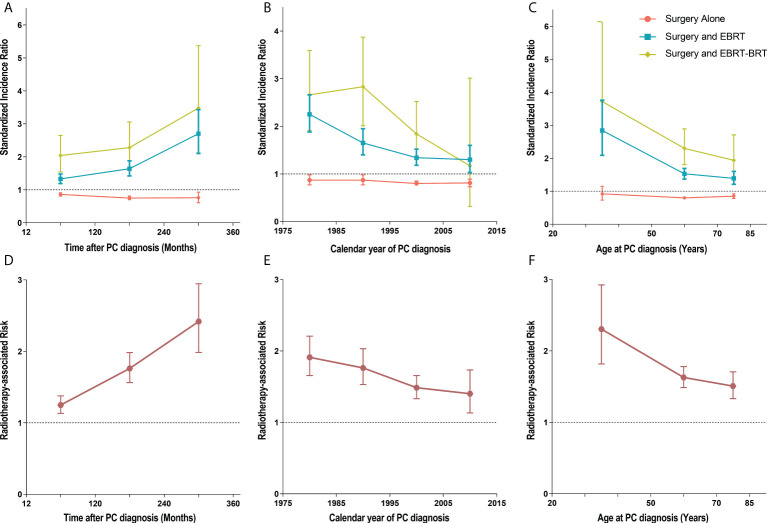
**(A)** Dynamic standardized incidence ratio (SIR) for secondary bladder cancer (SBC) in time after PC diagnosis (latency period)-SIR plot; **(B)** Dynamic SIR for SBC in calendar year at primary PC diagnosis-SIR plot; **(C)** Dynamic SIR for SBC in age at PC diagnosis-SIR plot; **(D)** Dynamic radiotherapy-associated risk (RR) for SBC in time after PC diagnosis (latency period)-RR plot; **(E)** Dynamic RR for SBC in calendar year at PC diagnosis-RR plot; **(F)** Dynamic RR for SBC in age at PC diagnosis-RR plot. **(A–C)** SIR of developing SBC in patients treated with radiation therapy (RT) versus the US general population are plotted, as well as patients treated without RT versus the US general population, and the incidence in the background US population is represented by the gray line. **(D–F)** RR was calculated by Poisson regression analysis with the relative risk of SBC development for PC patients receiving radiotherapy compared with those no receiving radiotherapy. This detailed data of SIR be shown in the **Table 3**, **Supplementary Table S3**. PC, pelvic cancer; SBC, secondary bladder cancer; SIR, standardized incidence ratio; RR, radiotherapy-associated risk; RT, radiation therapy; EBRT, external beam radiation therapy; EBRT–BRT, external beam radiation therapy with brachytherapy involving implants or isotopes.

To estimate the dynamic incidence risk associated with radiotherapy of developing SBC, we performed three dynamic SIR plots and three dynamic RR plots according to the time after PC diagnosis (latency period), year at primary PC diagnosis and age at primary PC diagnosis. From the dynamic SIR plots, the incidence of SBC in PC patients receiving radiotherapy was higher than that in the US general population (**Figures 4A–C**). In addition, we generated dynamic RR plots according to a similar tendency of SIR that could also be observed in SBC patients (**Figures 4D–F**). In the dynamic latency-SIR plot and RR plot, the risk of SBC increased as the latency time went on in PC patients, and risk significantly increased in the late latency, which was similar to the RR plot (**Figures 4A, D**). In the dynamic diagnosis year-SIR plot and RR plot, a decreasing tendency of risk could be observed in primary PC patients from 1975-2015 **(** **Figures 4B, E** **)**. This may be due to the improvement of radiotherapy technology, which makes the treatment process more precise and avoids the occurrence of excessive radiotherapy. In the dynamic age-SIR plot and RR plot, the increasing risk of SBC could be noted at different ages in the PC cancer diagnosis group undergoing RT. Compared with the US general population in the matching age group, PC patients with RT were at a significantly higher risk of developing SBC, especially younger PC patients, and younger patients had a higher incidence of SBC than older patients (**Figures 4C, F**). The detailed SIR and Poisson regression data are shown in **Table 3** and **Supplementary Table S3**.

**Table 3:** Standardized incidence ratio (SIR) of SBC.

Characteristic	NRTSIR (95% CI)	EBRTSIR (95% CI)	EBRT–BRTSIR (95% CI)
**ALL**	0.82 (0.78-0.85) ^#^	1.53 (1.42-1.66) ^#^	2.29 (1.90-2.73) ^#^
**Age at PC diagnosis**			
20-49	0.92 (0.73-1.15)	2.84 (2.09-3.76) ^#^	3.72 (2.08-6.14) ^#^
50-69	0.80 (0.76-0.84) ^#^	1.53 (1.37-1.69) ^#^	2.30 (1.81-2.89) ^#^
70-84	0.85 (0.78-0.92) ^#^	1.39 (1.21-1.60) ^#^	1.94 (1.34-2.71) ^#^
**Year of PC diagnosis**			
1975-1984	0.87 (0.77-0.98) ^#^	2.25 (1.88-2.66) ^#^	2.66 (1.91-3.59) ^#^
1985-1994	0.87 (0.77-0.98) ^#^	1.65 (1.40-1.95) ^#^	2.83 (2.01-3.87) ^#^
1995-2004	0.80 (0.76-0.85) ^#^	1.34 (1.18-1.52) ^#^	1.84 (1.30-2.52) ^#^
2005-2015	0.81 (0.73-0.89) ^#^	1.30 (1.03-1.60) ^#^	1.17 (0.32-3.01)
**Sex**			
Female	0.91 (0.82-0.99) ^#^	2.18 (1.91-2.48) ^#^	2.58 (2.11-3.13) ^#^
Male	0.80 (0.76-0.84) ^#^	1.30 (1.17-1.44) ^#^	1.38 (0.82-2.18)
**Race**			
White	0.80 (0.77-0.84) ^#^	1.52 (1.40-1.65) ^#^	2.24 (1.84-2.70) ^#^
Black	1.00 (0.83-1.20)	1.81 (1.19-2.64) ^#^	1.77 (0.65-3.84)
Other	1.02 (0.80-1.29)	1.56 (1.04-2.25) ^#^	5.18 (2.24-10.21) ^#^
**Tumor site**			
Rectum and Rectosigmoid	0.93 (0.84-1.02)	1.34 (1.18-1.52) ^#^	0.95 (0.02-5.28)
Cervix uteri	1.27 (0.93-1.69)	3.41 (1.99-5.46) ^#^	3.38 (2.17-5.03) ^#^
Corpus uteri	0.85 (0.75-0.96) ^#^	2.12 (1.79-2.50) ^#^	2.40 (1.91-2.99) ^#^
Ovary	0.89 (0.63-1.21)	2.82 (1.13-5.81) ^#^	0.00 (0.00-74.42)
Prostate	0.77 (0.73-0.81) ^#^	1.40 (1.21-1.62) ^#^	1.49 (0.88-2.36)
Anus, anal canal and anorectum	1.07 (0.55-1.87)	1.53 (0.89-2.45)	0.00 (0.00-23.98)
**Latency**			
12-119 months	0.86 (0.81-0.91) ^#^	1.33 (1.19-1.48) ^#^	2.04 (1.54-2.65) ^#^
120-239 months	0.75 (0.70-0.81) ^#^	1.64 (1.42-1.88) ^#^	2.28 (1.66-3.06) ^#^
240-360 months	0.76 (0.61-0.93) ^#^	2.70 (2.10-3.43) ^#^	3.48 (2.12-5.37) ^#^

PC, pelvic cancers; SBC, secondary bladder cancer; SIR, standardized incidence ratios; CI, confidence interval; NRT, no radiation therapy; EBRT: external beam radiation therapy; EBRT–BRT: external beam radiation therapy with brachytherapy involving implants or isotopes.

^#^p < 0.05.

### Survival outcome of SBC

**Figure 5:**
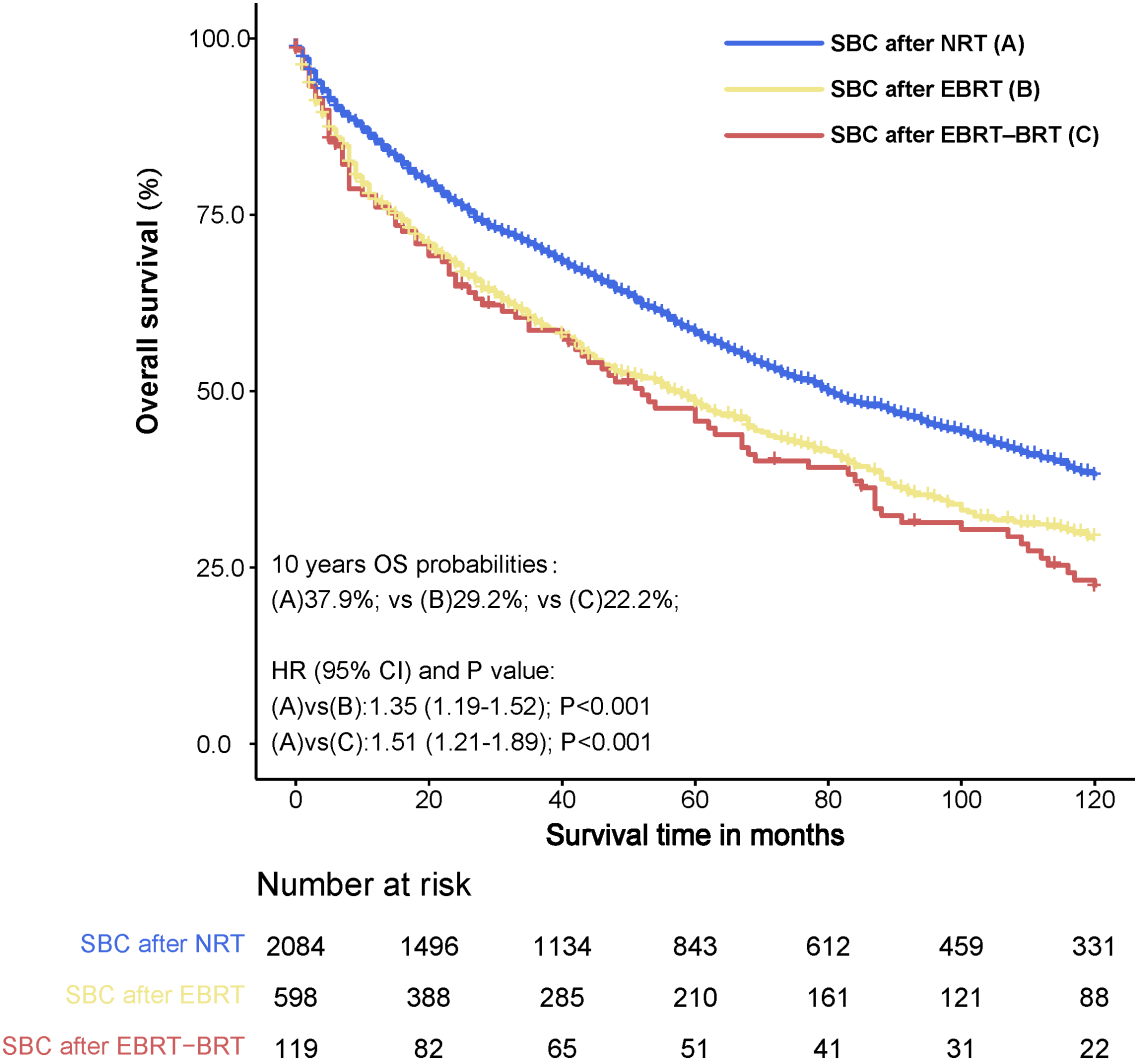
Survival comparison between primary pelvic cancer (PC) patients who developed secondary bladder cancer (SBC) after radiation therapy (RT) and no radiation therapy (NRT). Data. HRs were calculated using Cox regression. PC, pelvic cancers; SBC, secondary bladder cancer; HR, hazard ratio; CI, confidence interval; NRT, no radiation therapy; RT, radiation therapy; EBRT, external beam radiation therapy; EBRT–BRT, external beam radiation therapy with brachytherapy involving implants or isotopes.

For further analysis of the effect of radiotherapy on the survival of SBC, we compared survival between PC patients undergoing radiotherapy and those not undergoing radiotherapy. The 10-year survival rates of SBC after NRT, SBC after EBRT and SBC after EBRT-BRT were 37.9%, 29.2%, and 22.2%, respectively. There were significant differences between the 10-year OS of patients developing SBC after radiotherapy and that of patients not receiving radiotherapy (NRT vs. EBRT, adjusted HR= 1.35, 95% CI: 1.19-1.52, P<0.001; NRT vs. EBRT–BRT, adjusted HR= 1.51, 95% CI: 1.21-1.89, P<0.001) (**Figure 5**).

## Discussion

SPM in cancer survivors account for a significant proportion of the total cancer incidence, ranging from 11% to 25% in adults (15, 16). The development of SPM is associated with several important risk factors, including genetic background, lifestyle, environmental factors, and cancer-related treatment of first primary malignancies (14, 17–19). RT plays a substantial role in the development of SPM, and SPM associated with RT are a severe complication of cancer treatment (20–23).

This study was a large-scale population-based study that comprehensively assessed the risk of SBC in PC survivors and the survival outcome of SBC. We found that the cumulative incidence of SBC in PC patients who received RT was higher than that in patients without RT. In addition, the incidence of SBC in PC patients receiving RT was higher than that of the entire American population, and the risk of SBC after RT increased with the latency period. Younger patients receiving RT were more likely to develop SBC. In sum, pelvic RT is associated with a potentially increased risk for secondary tumors. In particular, the bladder, which is in close anatomical relation to several pelvic target organs, is likely to be in the radiation range and can consequently receive relatively high doses of radiation.

This is the first study using a large population-based cohort to conduct a comprehensive investigation of the prognosis of bladder cancer as the second primary malignancy, revealing a distinct, time‐varying disease course. Our research has the following advantages. First, the findings were based on the SEER database. A total of 318,165 pelvic tumors were included to avoid the selection bias imposed by single-center studies or small-sample studies. Second, the research period was 40 years, which made the conclusions more reliable. Third, competing-risk proportional hazard regression was used to obtain unbiased estimates of the risk factors for SBC. The Kaplan–Meier method and the Cox proportional approach are the main analysis methods of traditional risk prediction models, which can only manage one result and may produce biased results in the presence of competitive risks. However, in our study, the risk of competing is particularly important because a considerable portion of PC survivors usually die for other reasons before the development of SBC.

According to the SIR and Poisson regression analysis, we have the following main findings. First, the risk of SBC after radiation therapy decreases with the year of diagnosis, which may be related to the type of radiation therapy they receive. Technical improvements in radiotherapy over the last 30 years have also reduced its side effects on patients. Second, the risk of developing SBC after radiation therapy increases with increasing latency, which may be due to increased screening, improved medical imaging, and the development of treatment strategies that may have significantly increased detection rates and prolonged patient survival, resulting in a higher risk of exposure to subsequent cancers. Third, the risk of SBC after radiotherapy decreases with increasing age at the time of primary cancer diagnosis, which may be because younger patients treated with RT have more prolonged survival and are more likely to develop SBC. Our preliminary findings may help clinicians better understand SPM, and the results suggest that prolonged follow-up is needed for patients treated with pelvic RT, especially younger patients. When tumors occur more than 10 years later, patients are more likely to develop SBC (24).

However, at the same time, our study had some limitations. First, only the initial treatment information of the tumor was recorded in the SEER database, and it was unknown whether delayed RT was performed in the subsequent treatment. Therefore, this may lead to an underestimation of the actual risk of SBC associated with RT. Second, our study cohort was collected retrospectively over a long period of time, during which treatment of the tumors improved considerably. The radiotherapy regimen for each patient was unavailable, which may have influenced the obtainment of more detailed conclusions. Third, family history, smoking status, and weight were the strongest cancer risk factors, but relevant information was unavailable from the SEER database.

## Conclusion

Radiotherapy for primary PC was associated with higher risks of developing SBC than patients unexposed to radiotherapy. Different pelvic RT treatment modalities had different effects on the risk of SBC. We suggest that patients with pelvic RT, especially young patients, require long-term monitoring of the risk of SBC.

## Data availability statement

The original contributions presented in the study are included in the article/**Supplementary Material**. Further inquiries can be directed to the corresponding authors.

## Author contributions

Conception and design: SL, XG, XW, ZJ. Collection and assembly of data: SL, RW, GY, HL, TC. Data analysis and interpretation: SL, RW, XG, XW, ZJ. Manuscript writing, final approval of manuscript, accountable for all aspects of the work: All authors. All authors contributed to the article and approved the submitted version.

## Conflict of interest

The authors declare that the research was conducted in the absence of any commercial or financial relationships that could be construed as a potential conflict of interest.

## Publisher’s note

All claims expressed in this article are solely those of the authors and do not necessarily represent those of their affiliated organizations, or those of the publisher, the editors and the reviewers. Any product that may be evaluated in this article, or claim that may be made by its manufacturer, is not guaranteed or endorsed by the publisher.
