# Supplementary material for: Risk and prognosis of secondary bladder cancer after radiation therapy for pelvic cancer
Source: Front Oncol. 2022 Aug 24;12:982792. doi: 10.3389/fonc.2022.982792 (PMC9449132; doi:10.3389/fonc.2022.982792)
Supplement: Supplementary file 1 [file Table_1.docx]

**Supplementary Tables**

**Supplementary Table 1.** Comparison of the characteristics of second bladder cancer (SBC) patients receiving radiotherapy (RT) or no radiation therapy (NRT).

| **Characteristic** | **NRT**  **(N=2084)** | **EBRT**  **(n=598)** | **EBRT–BRT**  **(n=119)** | **P-value** |
| --- | --- | --- | --- | --- |
| **Age at PC diagnosis, No. (%)** |  |  |  | <0.001 |
| 20-49 | 9 (0.43) | 7 (1.17) | 7 (5.88) |  |
| 50-69 | 610 (29.27) | 162 (27.09) | 31 (26.05) |  |
| 70-84 | 1465 (70.30) | 429 (71.74) | 81 (68.07) |  |
| **Year of PC diagnosis, No. (%)** |  |  |  | <0.001 |
| 1975-1984 | 61 (2.93) | 19 (3.18) | 7 (5.88) |  |
| 1985-1994 | 172 (8.25) | 79 (13.21) | 26 (21.85) |  |
| 1995-2004 | 493 (23.66) | 175 (29.26) | 35 (29.41) |  |
| 2005-2015 | 1358 (65.16) | 325 (54.35) | 51 (42.86) |  |
| **Sex, No. (%)** |  |  |  | <0.001 |
| Female | 415 (19.91) | 219 (36.62) | 101 (84.87) |  |
| Male | 1669 (80.09) | 379 (63.38) | 18 (15.13) |  |
| **Race, No. (%)** |  |  |  | 0.356 |
| White | 1896 (90.98) | 546 (91.30) | 107 (89.92) |  |
| Black | 117 (5.61) | 26 (4.35) | 5 (4.20) |  |
| Other | 71 (3.41) | 26 (4.35) | 7 (5.88) |  |
| **Tumor grade, No. (%)** |  |  |  | 0.673 |
| Grade I/II | 840 (40.31) | 256 (42.81) | 47 (39.50) |  |
| Grade III/IV | 899 (43.14) | 238 (39.80) | 53 (44.54) |  |
| Unknow | 345 (16.55) | 104 (17.39) | 19 (15.97) |  |
| **Tumor stage, No. (%)** |  |  |  | 0.028 |
| Localized | 1528 (73.32) | 411 (68.73) | 82 (68.91) |  |
| Regional | 312 (14.97) | 96 (16.05) | 19 (15.97) |  |
| Localized/regional (Prostate cases) | 70 (3.36) | 38 (6.35) | 8 (6.72) |  |
| Unknown | 174 (8.35) | 53 (8.86) | 10 (8.40) |  |
| **Tumor size, No. (%)** |  |  |  | <0.001 |
| <5 cm | 201 (9.64) | 52 (8.70) | 8 (6.72) |  |
| ≥5 cm | 359 (17.23) | 61 (10.20) | 10 (8.40) |  |
| Unknown | 1524 (73.13) | 485 (81.10) | 101 (84.87) |  |
| **Surgery, No. (%)** |  |  |  | 0.586 |
| No | 1905 (91.41) | 550 (91.97) | 106 (89.08) |  |
| Yes | 179 (8.59) | 48 (8.03) | 13 (10.92) |  |
| **Chemotherapy, No. (%)** |  |  |  | 0.015 |
| No | 1678 (80.52) | 511 (85.45) | 101 (84.87) |  |
| Yes | 406 (19.48) | 87 (14.55) | 18 (15.13) |  |
| **Radiation, No. (%)** |  |  |  | 0.003 |
| No | 1970 (94.53) | 584 (97.66) | 116 (97.48) |  |
| Yes | 114 (5.47) | 14 (2.34) | 3 (2.52) |  |

**Abbreviations:** PC, pelvic cancers; SBC, secondary bladder cancer; NRT, no radiation therapy; RT, radiation therapy; EBRT, external beam radiation therapy; EBRT–BRT, external beam radiation therapy with brachytherapy involving implants or isotopes.

**Supplementary Table 2.** Univariable and multivariable Cox regression analysis of risk of developing secondary bladder cancer (SBC) in patients with pelvic cancers (PC).

| **Characteristic** | **Univariable analysis** | | **Multivariable analysis** | |
| --- | --- | --- | --- | --- |
|  | HR (95% CI) | P-value | HR (95% CI) | P-value |
| **Age at PC diagnosis** |  |  |  |  |
| 20-49 | Ref |  | Ref |  |
| 50-69 | 4.68 (3.94-5.56) | <0.001 | 3.30 (2.73-3.98) | <0.001 |
| 70-84 | 9.20 (7.67-11.03) | <0.001 | 6.81 (5.60-8.29) | <0.001 |
| **Year of PC diagnosis** |  |  |  |  |
| 1975-1984 | Ref |  | Ref |  |
| 1985-1994 | 1.09 (0.95-1.25) | 0.217 | 1.01 (0.88-1.16) | 0.876 |
| 1995-2004 | 1.86 (1.65-2.09) | <0.001 | 0.97 (0.83-1.12) | 0.631 |
| 2005-2015 | 1.39 (1.21-1.61) | <0.001 | 0.76 (0.65-0.90) | 0.002 |
| **Sex** |  |  |  |  |
| Female | Ref |  | Ref |  |
| Male | 3.62 (3.32-3.95) | <0.001 | 3.42 (2.86-4.08) | <0.001 |
| **Race** |  |  |  |  |
| White | Ref |  | Ref |  |
| Black | 0.63 (0.54-0.75) | <0.001 | 0.62 (0.52-0.73) | <0.001 |
| Other | 0.50 (0.41-0.61) | <0.001 | 0.54 (0.45-0.66) | <0.001 |
| **Tumor grade** |  |  |  |  |
| Grade I/II | Ref |  | Ref |  |
| Grade III/IV | 1.29 (1.18-1.41) | <0.001 | 1.16 (1.06-1.27) | 0.001 |
| Unknow | 0.59 (0.52-0.67) | <0.001 | 1.03 (0.89-1.18) | 0.718 |
| **Tumor stage** |  |  |  |  |
| Localized | Ref |  | Ref |  |
| Regional | 1.92 (1.71-2.16) | <0.001 | 1.09 (0.96-1.24) | 0.180 |
| Localized/regional (Prostate cases) | 2.64 (2.42-2.88) | <0.001 | 1.03 (0.90-1.19) | 0.643 |
| **Tumor size** |  |  |  |  |
| <5 cm | Ref |  |  |  |
| ≥5 cm | 0.82 (0.64-1.06) | 0.132 | - | - |
| Unknown | 1.14 (0.94-1.38) | 0.189 | - | - |
| **Tumor site** |  |  |  |  |
| Rectum and rectosigmoid | Ref |  | Ref |  |
| Cervix uteri | 0.22 (0.17-0.27) | <0.001 | 1.08 (0.81-1.44) | 0.591 |
| Corpus uteri | 0.41 (0.37-0.46) | <0.001 | 0.94 (0.78-1.14) | 0.534 |
| Ovary | 0.21 (0.16-0.29) | <0.001 | 0.81 (0.58-1.13) | 0.209 |
| Prostate | 1.24 (1.13-1.36) | <0.001 | NA | NA |
| Anus, anal canal and anorectum | 0.79 (0.55-1.15) | 0.215 | 0.99 (0.68-1.44) | 0.960 |
| **Chemotherapy** |  |  |  |  |
| No | Ref |  |  |  |
| Yes | 1.00 (0.88-1.14) | 0.996 | - | - |
| **Radiation** |  |  |  |  |
| No | Ref |  | Ref |  |
| EBRT | 1.70 (1.56-1.86) | <0.001 | 1.75 (1.59-1.94) | <0.001 |
| EBRT–BRT | 1.30 (1.08-1.56) | <0.001 | 2.33 (1.91-2.84) | <0.001 |

**Abbreviations:** PC, pelvic cancers; SBC, secondary bladder cancer; HR, hazard ratio; CI, confidence interval; EBRT, external beam radiation therapy; EBRT–BRT, external beam radiation therapy with brachytherapy involving implants or isotopes.

**Supplementary Table 3.** Risk of developing secondary bladder cancer (SBC) in patients with pelvic cancers (PC) by Poisson regression analysis

| **Characteristic** | **Univariable analysis** | | **Multivariable analysis** | |
| --- | --- | --- | --- | --- |
|  | RR (95% CI) | P-value | RR (95% CI) | P-value |
| **ALL** | 1.34 (1.26-1.43) | <0.001 | 1.63 (1.52-1.75) | <0.001 |
| **Age at PC diagnosis** |  |  |  |  |
| 20-49 | 2.17 (1.73-2.71) | <0.001 | 2.30 (1.82-2.92) | <0.001 |
| 50-69 | 1.32 (1.22-1.44) | <0.001 | 1.63 (1.49-1.78) | <0.001 |
| 70-84 | 1.23 (1.09-1.39) | 0.001 | 1.51 (1.33-1.71) | <0.001 |
| **Year of PC diagnosis** |  |  |  |  |
| 1975-1984 | 1.65 (1.44-1.89) | <0.001 | 1.91 (1.66-2.21) | <0.001 |
| 1985-1994 | 1.70 (1.49-1.94) | <0.001 | 1.76 (1.53-2.03) | <0.001 |
| 1995-2004 | 1.20 (1.09-1.33) | <0.001 | 1.49 (1.33-1.66) | <0.001 |
| 2005-2015 | 1.15 (0.94-1.40) | 0.188 | 1.40 (1.13-1.73) | 0.002 |
| **Sex** |  |  |  |  |
| Female | 1.88 (1.71-2.06) | <0.001 | 1.81 (1.65-1.99) | <0.001 |
| Male | 1.50 (1.35-1.66) | <0.001 | 1.46 (1.32-1.62) | <0.001 |
| **Race** |  |  |  |  |
| White | 1.35 (1.26-1.44) | <0.001 | 1.64 (1.53-1.77) | <0.001 |
| Black | 1.15 (0.85-1.55) | 0.376 | 1.25 (0.90-1.72) | 0.176 |
| Other | 1.54 (1.13-2.08) | 0.006 | 1.86 (1.33-2.60) | <0.001 |
| **Tumor site** |  |  |  |  |
| Rectum and rectosigmoid | 1.27 (1.09-1.49) | 0.002 | 1.37 (1.16-1.61) | <0.001 |
| Cervix uteri | 2.08 (1.64-2.64) | <0.001 | 1.84 (1.44-2.36) | <0.001 |
| Corpus uteri | 1.84 (1.64-2.06) | <0.001 | 1.73 (1.54-1.95) | <0.001 |
| Ovary | 3.43 (1.61-7.32) | 0.001 | 2.78 (1.25-6.19) | 0.012 |
| Prostate | 1.80 (1.58-2.04) | <0.001 | 1.63 (1.43-1.85) | <0.001 |
| Anus, anal canal and anorectum | 0.91 (0.44-1.89) | 0.801 | 1.07 (0.49-2.31) | 0.873 |
| **Latency** |  |  |  |  |
| 12-119 months | 1.15 (1.05-1.26) | 0.002 | 1.25 (1.13-1.38) | <0.001 |
| 120-239 months | 1.56 (1.40-1.74) | <0.001 | 1.76 (1.56-1.98) | <0.001 |
| 240-360 months | 2.23 (1.85-2.69) | <0.001 | 2.42 (1.98-2.95) | <0.001 |

**Abbreviations:** PC, pelvic cancers; SBC, secondary bladder cancer; RR, radiotherapy-associated risk; CI, confidence interval.
